# Determinants of exclusive breastfeeding practice in Bangladesh: Evidence from nationally representative survey data

**DOI:** 10.1371/journal.pone.0236080

**Published:** 2020-07-15

**Authors:** Md. Aminur Rahman, Md. Nuruzzaman Khan, Shahinoor Akter, Azizur Rahman, Md. Mahmudul Alam, Md. Alam Khan, Md. Mostafizur Rahman

**Affiliations:** 1 Department of Population Science and Human Resource Development, University of Rajshahi, Rajshahi, Bangladesh; 2 Department of Population Sciences, Jatiya Kabi Kazi Nazrul Islam University, Mymensingh, Bangladesh; 3 Department of Anthropology, Jagannath University, Dhaka, Bangladesh; 4 Department of Public Administration and Governance Studies, Jatiya Kabi Kazi Nazrul Islam University, Mymensingh, Bangladesh; 5 Department of Statistics, University of Rajshahi, Rajshahi, Bangladesh; 6 Department of Pharmacy, University of Rajshahi, Rajshahi, Bangladesh; Institute of Economic Growth, INDIA

## Abstract

**Background:**

Exclusive breastfeeding (EBF) means that an infant should be breastfed only for the first six months of life to achieve optimal child development and to prevent infant morbidity and mortality. The aim of this analysis was to determine the individual-, household-, and community-level factors associated with EBF practice in Bangladesh.

**Methods:**

A total of 1,440 women-child pairs data were analysed extracted from 2011 and 2014 Bangladesh Demographic and Health Survey. Multilevel logistic regression models were used separately for individual-, household-, and community level factors to identify the different level of factors associated with EBF practice.

**Results:**

Around 61% women in Bangladesh practiced EBF with significant variation across several individual-, household-, and community-level factors. At the individual level, higher odds of EBF practice was found among mothers' received higher number of antenatal care and lower age of child. Mothers' higher education and engagement in formal jobs were found negatively associated with EBF practice. At the community level, higher odds of EBF was found among women live in Barishal, Dhaka, and Rajshahi divisions, and resided in the community with moderate level of female education, higher level of fertility, and higher use of antenatal and delivery care.

**Conclusions:**

One in every three children in Bangladesh do not breastfeed exclusively which needs special attention for the policymakers. In this case, educated women engaged in income generating activities and women did not use antenatal care should be given priority. At the community level, priority should be given for the women's resides in the community with lower level of antenatal and delivery healthcare services use.

## Introduction

Exclusive breastfeeding (EBF) up to six months offers incredible health benefits to both the infant and mother, and protect the child health by providing required nutritional needs for the infants for the first six months of life [1, 2]. Moreover, this practice substantially reduces the risk of infant morbidity and mortality through eliminates the risk of contamination from formula milk and other fluids and foods, and ensure proper early childhood development, including mental and motor development [3–5]. Considering these remarkable immunological and anti-inflammatory properties that protect both mother and children against various infections and diseases, in 2001, the World Health Organization (WHO) approved a guideline for EBF practice where emphasis was made that a child should be breastfed exclusively for the first six months of age, and afterward, breastfeeding with appropriate complementary foods up to two years of age [6]. Although this guideline along with universal awareness have been contributed to a substantial increase in EBF practice worldwide, this rate is still lower in developing countries, 50% [7–10].

Trend to increase EBF practice has recently been slowing, though it is adapted as one of the six global nutritional targets to be achieved by 2025, and riding as core in global maternal and child health agendas [11]. Consequently, international organisations such as the World Bank, United Nations Children's Fund (UNICEF), and the WHO are now prioritising challenges and supporting for funding and making public-private partnership to increase the rate of EBF particularly in Low and Lower-Middle Income Countries (LLMICs) [11, 12]. The targets for these programs are to ensure breastfeeding up to the second year of life and scaling up nutrition [11, 12]. This implies that extensive work and continued efforts are inevitable as underlying reasons for lower EBF practice are multifaceted and complex [9, 13].

Practising EBF for six months might be difficult for mothers, particularly in LLMICs, including Bangladesh, where maternal malnutrition is common [14, 15]. Besides, lack of information on benefits of EBF practice, inadequate assistance to mother in the workplace and insufficient support provided by the healthcare system contribute to discontinue breastfeeding before the recommended duration of six months [2]. Moreover, evidence shows that several socio-demographic factors play critical roles in practising EBF among women in LLMICs. Studies conducted in Bangladesh, Brazil and Malaysia found that factors like lower maternal age, lower level of schooling, and lower-income status were positively associated with the lower rate of EBF practice [16–18]. Birth characteristics including vaginal delivery and adequate counselling on infant feeding were the factors found associated with increased odds of EBF practise in an Ethiopian's study [19]. Authors of that Ethiopian's study also emphasised the role of prenatal EBF plan and found the strongest significant effect on the increasing duration of EBF. Child factors such as child age less than three months and female gender were found as positive predictors of EBF in two studies conducted in Nigeria [20], and Ethiopia [21]. Healthcare services utilisation including antenatal care (ANC), delivery care, and postnatal care services, were also found as significant positive predictors of EBF practice in multiple studies [21, 22].

Above mentioned studies have had few similar limitations. For instance, all studies included individual-level characteristics only as factors that influencing EBF practice. Moreover, authors of these studies used single-level analytical techniques that did not consider clustering and hierarchical structure of data that arise for individuals living in different communities, cities or enumerations areas. Therefore, this sort of analysis in population-level data is misleading in various ways. For example, when the cluster or community-level factors are ignored while analysing data, it might lead to violation of the assumption of independency between observations in a group that underestimate the standard error and produce significant false results [23].

Moreover, previous studies found the sample from particular contexts such as cluster or community share same physical and socio-economic characteristics and establish social networks that influence individual health behaviours, therefore need to be considered for getting accurate results [24, 25]. Consequently, this analysis aimed to determine individual-, household-, and community-level factors associated with the EBF practice up to six months in Bangladesh. Data from two nationally representative cross-sectional Bangladesh Demographic and Health Surveys (BDHS) conducted in 2011 and 2014 were analysed through use of multilevel logistic regression model by considering community-level random intercept.

## Materials and methods

### Ethical issue

Data used in this study was obtained from MEASURE DHS archive collected by Macro, Calverton, USA. The ORC Macro Institutional Review Board and Bangladesh Medical Research Council reviewed and approved these survey and data collection procedure.

### Sources of data

This study used two consecutive nationally representative cross-sectional BDHSs data collected in 2011 and in 2014. Description regarding each of the surveys is published elsewhere [2, 26]. The National Institute of Population Research and Training (NIPORT) conducted these surveys monitored by the Ministry of Health and Family Welfare in Bangladesh. Technical and financial supports were provided by the ICF International of Calverton, Maryland, USA, and the USAID respectively. Each survey was based on households data of reproductive-aged adults women (15–49 years old) collected through the two-stage cluster sampling method. At the first stage, 600 primary sampling units (clusters) were selected covering each administrative divisions, and rural and urban areas separately. For this, most recent census enumeration areas prepared by the Bangladesh Bureau of Statistics during 2011 national population census was used for both surveys. At the second stage of sampling, 30 households were selected from each cluster with the systematic random sampling method, and each married woman aged 15–49 years old in the selected households were interviewed. A sub-sample of these interviewed women met the inclusion criteria were analysed. The inclusion criteria were (i) women who had at least one child aged 0–6 months (ii) responded to the questions on breastfeeding and supplementary feeding status.

### Exposure variable

Status of EBF from birth to six months of the alive child was considered as an exposure variable. This variable was created based on respondents' responses on the following questions; (i) whether the baby was still being breastfed, (ii) the duration of breastfeeding, and (iii) if other foods were given during the last 24 hours at the time survey. These questions were asked following women's response to having a child aged 2 years or less. We categorised sample as exclusive breastfeeding practicing mothers if reported continued breastfeeding up to six months with no other foods and liquids (1), otherwise categorised as not exclusively breastfeeding practising mothers (0).

### Covariates

Different individual-, household- and community- level characteristics were considered as covariates. Individual-level characteristics were mother's age (≤19 years, 20–34 years, ≥35 years), mother's educational status (illiterate, primary, secondary, higher), mother's occupation (not working, any form of formal working), and child age (0–2 months, 3–4 months, 5–6 months). Other individual-level factors considered were health care services level factors: number of ANC visits (no visit; 1–3 visits; ≥4 visits), place of delivery (home; health care institution), and postnatal care service visit (no visit; at least one visit). Wealth index, partner's educational status (illiterate; primary; secondary; higher), partner's occupation (agricultural worker; services and non-agricultural labor; business and others), and number of children ever born (≤2; 3–4; >4) were considered as household-level characteristics. The wealth index variable in BDHSs included was calculated based on principal component analysis of the questions related to the household wealth. In the original survey, this variable was classified with five quintiles (poorest; poorer; middle; richer; richest) which was recoded as richer (richest; richer), middle, and poorer (poorest; poorer) in this study.

Place of residence (urban; rural), and place of region (Barishal; Chattogram; Dhaka; Khulna; Rajshahi; Rangpur; Sylhet) were considered as community-level characteristics. Other community-level characteristics that were included are not directly available in the datasets used. Hence, constructed by aggregating the individual-, and household- level characteristics at the clusters' level. These included community-level illiteracy (low; medium; high), community-level poverty (low; medium; high), and community level fertility (low; high). Cluster level aggregate data on illiteracy (no education), poverty (poorer and poorest in wealth quintile), and fertility (children ever born) were used to generate these variables. The last three community-level characteristics were considered as community-level ANC service use, community-level delivery care services use, and community level postnatal health care services use. These were the aggregated values of community-level use of the respective services measured from the individual level data on healthcare services use and categorised as low use areas (the proportion was between 0 and 45%), and high use areas (the proportion was between 46 and 100%).

### Statistical analysis

Descriptive statistics with survey weight were used to describe the characteristics of the respondents. Bivariate analysis was used to see the percentage of EBF practice across selected individual-, household-, and community-level factors. In BDHSs data, individuals included from a community were more likely share similar lifestyles, and use a similar healthcare facility. Therefore, it is expected that individual response from the same cluster would behave alike from different clusters. For such type of cluster data, multilevel logistic regression model produces better results. Therefore, both the unadjusted and adjusted multilevel logistic regression models were used to assess the association between EBF and individual-, household-, and community-level characteristics. In the unadjusted model, EBF practice was considered with particular individual-, household-, and community-level characteristics. Two adjusted models were run separately for individual- and household- level characteristics (controlled of community-level characteristics) and community-level characteristics (controlled of individual and household-level characteristics). All analyses were conducted by using Stata software version 15.1/MP (Stata Corp, College Station, Texas, USA).

## Results

Total of 1440 women-child pairs data were analysed extracted from BDHS 2011 (798), and 2014 (642). The background characteristics of the mothers and children are presented in Table 1. The mean age of the mothers was 23 years. Nearly 84% of the total mothers was formally educated (primary or above) and not engaged with any formal jobs (85%). The mean age of the children was 2.05 months and around half of them was female (48%). Table 2 shows a decreased prevalence of EBF from 65% to 56% during the survey years 2011 and 2014. We found a higher prevalence of EBF practice among mothers aged 20–34 years (62%), having higher education (66%), higher educated partners (64%), higher in socio-economic status (63%), and delivered in healthcare facilities (64%). Differences in the prevalence of EBF were also found across regions of residence where the women's residing in, and other community-level characteristics such as rates of community-level female education, community-level poverty, community-level fertility, and community level services utilisation (Table 3).

**Table 1:** Background characteristics of included mothers and their child, (N = 1440).

Characteristics	Mean (95% CI)/Prevalence (95% CI)
**Perinatal and Infant characteristics**	
Women's age at birth, mean	23.46 (23.29–23.62)
No formal occupation	85.25 (83.71–86.66)
Formally educated (primary or above)	84.80 (83.06–86.40)
Household poor wealth quintile	41.38 (39.14–43.66)
Household rich wealth quintile	38.60 (36.08–41.17)
Antenatal care received in number, mean	2.58 (2.47–2.69)
Child’s age in months, mean	2.05 (1.99–2.11)
Female child	48.06 (46.44–49.68)
Delivery by cesarean section	21.12 (19.63–22.69)
Low birth weight	27.08 (25.52–28.69)

**Table 2:** Bivariate analysis of exclusive breastfeeding practice by the individual, and household-level characteristics, BDHS 2011–2014 (All estimates are weighted).

Individual and household-level characteristics	Exclusive breastfeeding practice		Total
	Number	Percentage	
**Survey's year**			
2011	519	65	798
2014	359	56	642
**Mother's age (in years)//**			
≤19	238	59	406
20–34	615	62	984
≥35	25	50	50
**Mother's educational status**			
Illiterate	124	56	219
Primary ^1^	247	59	418
Secondary ^2^	416	63	664
Higher	92	66	139
**Partner's educational level**			
Illiterate	198	61	323
Primary ^1^	261	56	464
Secondary ^2^	297	64	463
Higher	122	64	190
**Wealth index**			
Poorer	341	59	579
Middle	182	61	301
Richer	355	63	560
**Place of delivery**			
Home	534	59	899
Health care institutions	344	64	541
**Antenatal visit (in times)**			
No visit	225	65	348
≤4	474	59	796
>4	179	61	296
**Child's Gender**			
Male	467	60	772
Female	411	62	668
**Child's Age**			
0–2 months	362	84	433
3–4 months	342	67	506
4–6 months	174	35	501
**Children ever born**			
≤2	611	61	1002
3–4	223	62	361
>4	44	57	77
**Partner's occupation**			
Agricultural worker	237	59	399
Services and non-agricultural labor	440	62	710
Business and others	201	61	331
**Mother's occupation**			
Not working	773	61	1275
Any form of formal work	105	64	165
**Total**	878	61	1440

**Table 3:** Bivariate analysis of exclusive breastfeeding practice by the community-level characteristics, BDHS 2011–2014 (All estimates are weighted).

Community-level characteristics	Exclusive breastfeeding practice		Total
	Number	Percentage	
**Place of residence**			
Rural	667	61	1091
Urban	211	60	349
**Region of residence**			
Barisal	35	44	79
Chittagong	230	67	345
Dhaka	235	52	457
Khulna	92	69	133
Rajshahi	97	59	165
Rangpur	107	73	146
Sylhet	82	71	115
**Community-level female education**			
Low (<25%)	376	64	587
Moderate (25%– 50%)	355	57	622
High (>50%)	147	63	231
**Community-level poverty**			
Low (<25%)	306	58	524
Moderate (25%– 50%)	277	60	461
High (>50%)	295	65	455
**Community-level fertility**			
Low fertility areas (TFR<2.10)	434	64	675
High fertility areas (TFR≥2.10)	444	58	765
**Community-level ANC use**			
Low use areas (0–49%)	143	67	212
High use areas (≥50%)	735	60	1228
**Community-level delivery care use**			
Low use areas (0–49%)	225	66	341
High use areas (≥50%)	653	59	1099
**Community-level postnatal care use**			
Low use areas (0–49%)	662	60	1097
High use areas (≥50%)	216	63	343
**Total**	878	61	1440

Table 4 presents the results of the multilevel logistic regression model to assess the association between EBF practice and individual- and household-level characteristics. Around 48% (aOR 1.477, 95% CI, 1.162–1.877, *p<0*.*01*) higher odds of EBF practise was found among the mothers from the survey conducted in 2014 as compared to the survey conducted in 2011. We found higher likelihoods of not practising EBF among secondary (aOR,0.659, 95% CI, 0.441–0.987, *p<0*.*05*) and higher educated mothers (aOR 0.520, 95% CI, 0.280–0.966, *p<0*.*05*) than illiterate mothers. Around 39% (aOR, 1.386, 95% CI, 1.027–1.872, *p<0*.*05*) and 58% (aOR, 1.581, 95% CI, 1.066–2.345, *p<0*.*05*) higher use of EBF practice were found among the mothers' used 1–3 and ≥4 ANC visits than mothers' who did not use ANC visit. We found the higher likelihoods of EBF practice for the children aged 0–2 months (aOR, 10.828, 95% CI, 7.750–15.185, *p<0*.*05*) and 3–4 months (aOR, 2.472, 95% CI, 1.748–3.413, *p<0*.*05*) than children aged 4–6 months. Around 31% (aOR, 0.698, 95% CI, 0.482–0.989, *p<0*.*05*) lower likelihood EBF practice was found among mothers' engaged in any form of formal work compared to mothers' who were not involved in any formal work. Among the individual- and household level factors included in the model, mother's age, partner's education, wealth index, place of delivery, postnatal care visit, child's gender, number of children ever born, and partner's education were not found associated with the EBF practice.

**Table 4:** Results of multilevel logistic regression analysis of individual-, and household- level characteristics (adjusted with community levecharecteristics) with EBF practice, 2011–2014.

Characteristics	Individual-, and household-level characteristics of EBF practice	
	Unadjusted Odds ratio (95% CI)	Adjusted Odds ratio** (95% CI)
**Survey's year**		
2011®	1.000	1.000
2014	1.453 (1.174–1.799)***	1.477 (1.162–1.877)***
**Mother's age (in years)**		
≤19®	1.000	1.000
20–34	0.850 (0.671–1.076)	0.919 (0.696–1.214)
>30	1.400 (0.778–2.520)	1.546 (0.781–3.059)
**Mother's educational status**		
Illiterate®	1.000	1.000
Primary ^1^	0.896 (0.643–1.247)	0.720 (0.495–1.046)*
Secondary ^2^	0.773 (0.567–1.053)	0.659k (0.441–0.987)**
Higher	0.671 (0.432–1.041)*	0.520 (0.280–0.966)lk**
**Partner's educational level**		
Illiterate®	1.00	1.00
Primary ^1^	1.235 (0.925–1.650)	1.267 (0.910–1.763)
Secondary ^2^	0.881 (0.657–1.182)	1.036 (0.715–1.499)
Higher	0.878 (0.605–1.273)	1.168 (0.698–1.957)
**Wealth index**		
Poorer®	1.000	1.000
Middle	0.930 (0.699–1.236)	0.916 (0.660–1.271)
Richer	0.826 (0.651–1.049)	0.881(0.631–1.101)
**Place of delivery**		
Home®	1.000	1.000
Health care institutions	0.839 (0.673–1.045)	0.834 (0.631–1.101)
**Antenatal visit (in times)**		
No visit®	1.000	1.000
1–3 visits	1.243 (0.957–1.614)	1.386 (1.027–1.872)**
≥4 visits	1.181 (0.857–1.628)	1.581 (1.066–2.345)**
**Postnatal care visit**		
No visits®	1.00	1.00
At least one visit	0.982 (0.751–1.795)	0.886 (0.741–1.861)
**Child's Gender**		
Male®	1.000	1.000
Female	0.957 (0.774–1.184)	0.955 (0.763–1.195)
**Child's age**		
4–6 months®	1.000	1.00
0–2 months	9.503 (6.945–13.004)***	10.828 (7.750–15.185)***
3–4 months	2.452 (1.790–3.360)***	2.472 (1.748–3.413)***
**Children ever born**		
≤2®	1.000	1.000
3–4	0.963 (0.752–1.234)	0.853 (0.626–1.164)
>4	1.186 (0.744–1.891)	1.028 (0.573–1.843)
**Partner's occupation**		
Agricultural worker®	1.000	1.000
Services and non-agricultural labor	0.894 (0.696–1.149)	0.865 (0.649–1.152)*
Business and others	0.940 (0.698–1.267)	1.014 (0.722–1.424)
**Mother's occupation**		
Not working®	1.000	1.000
Any form of formal work	0.868 (0.620–1.216)	0.698 (0.482–0.989)**

^1^Primary completed is defined as completing grade 5

^2^ Secondary completed is defined as completing grade 10

***P,0.01

** P<0.05

*P<0.10.

Table 5 shows the association between community-level characteristics and EBF practice. We found the region of residence was a significant predictor of practising EBF. Higher likelihoods of EBF practice was found among mothers living in Barishal (aOR 3.445, 95% CI 1.876–6.328, *p<0*.*01*), Dhaka (aOR, 2.447, 95% CI 1.540–3.888, *p<0*.*01*), and Rajshahi (aOR 1.938, 95% CI 1.165–3.225, *p<0*.*01*) regions than the mothers living in Rangpur region. Moderate community-level education (aOR 1.542, 95% CI 1.051–2.280, *p<0*.*05*), higher community level fertility (aOR, 1.347, 95% CI, 1.022–1.777, *p<0*.*05*), community-level high use of ANC (aOR, 1.441, 95% CI, 1.018–2.001, *p<0*.*05)* and delivery care (aOR, 1.369, 95% CI, 1.130–1.927, *p<0*.*05*) were the community-level factors found significantly associated with increased odds of EBF practice. However, no evidence of association of EBF practice with place of residence and community-level postnatal care services visit were found.

**Table 5:** Results of multilevel logistic regression analysis of community-level characteristics (adjusted with individual- and household level characteristics) with EBF practice in BDHS, 2011–2014.

Characteristics	Community-level characteristics of EBF practice	
	Unadjusted Odds ratio (95% CI)	Adjusted Odds ratio** (95% CI)
**Place of residence**		
Rural®	1.000	1.000
Urban	1.031 (0.805–1.319)	1.004 (0.739–1.364)
**Region of residence**		
Rangpur®	1.000	1.000
Barishal	3.352 (1.984–6.267)***	3.445 (1.876–6.328)***
Chattogram	1.361 (0.886–2.091)	1.273 (0.784–2.067)
Dhaka	2.571 (1.706–3.873)***	2.447 (1.540–3.888)***
Khulna	1.248 (0.743–2.095)	1.600 (0.913–2.805)
Rajshahi	1.916 (1.185–3.095)***	1.938 (1.165–3.225)**
Sylhet	1.112 (0.645–1.917)	0.826 (0.451–1.513)
**Community-level female education**		
High (>50%)®	1.000	1.000
Moderate (25%– 50%)	1.310 (0.967–1.776)*	1.542 (1.051–2.280) **
Low (<25%)	0.881 (0.632–1.227)	1.082 (0.692–1.693)*
**Community-level poverty**		
Low (<25%)®	1.000	1.000
Moderate (25%– 50%)	0.869 (0.685–1.102)	1.207 (0.878–1.661)
High (>50%)	0.918 (0.687–1.226)	1.436 (0.951–1.924)
**Community-level fertility**		
Low fertility areas®	1.000	1.000
High fertility areas	1.303 (1.053–1.612)**	1.347 (1.022–1.777)**
**Community-level ANC use**		
Low use areas®	1.000	1.000
High use areas	1.384 (1.016–1.885)**	1.441 (1.018–2.011)**
**Community-level delivery care use**		
Low use areas®	1.000	1.000
High use areas	1.365 (1.017–1.878)**	1.369 (1.130–1.927)**
**Community-level of postnatal care use**		
Low use areas®	1.000	1.000
High use areas	0.877 (0.675–1.140)	0.930 (0.731–1.183)**

* 10% level of significance ** 5% level of significance, ***1% level of significance, ‘®’ reference category’; ‘CI’ confidence interval.

*****P,0.01 ** P<0.05, *P<0.10.

## Discussion

This analysis investigates the individual-, household-, and community-level characteristics associated with the practising or not practising EBF. We found each level of characteristics affects EBF practice; however, this association found the strongest for community-level characteristics. At the individual level, mothers' higher education and engagement of formal works were found associated with lower odds of EBF practice. Higher use of ANC and lower age of children were found associated with increased odds of EBF. At the community level, region of residence, community-level moderate women's education, community-level higher fertility rate, community-level higher ANC, and delivery care services use were found to have a positive association with the increased odds of EBF practice.

Bangladesh observed a substantial increase in the EBF practice from 42% in 2004 to 64% in 2011 [27]; whereas, our analysis revealed a slight decline in the rate of EBF practice, nearly 61%. However, this estimate of EBF practice in Bangladesh was higher than other LLMICs like India (46.40%), Nepal (53.10%) [28], Malaysia (43.1%) [29], Ethiopia (46.5%) [30], and Uganda (50.00%) [31]. Different intensity of country-level policies to enhance EBF practice among women might contribute to such differences in EBF practice with cultural, economic, and socio-economic dissimilarities [9]. Infant and Young Child Feeding (IYCF) has always been a priority in Bangladesh national child health strategy to enhance child survival and reduce child malnutrition [2]. For this, the Government of Bangladesh (GoB) has implemented a round of national strategies of IYCFs, national health, population and nutrition sectors strategy plans, and five-yearly plans in collaboration with international agencies such as the WHO and UNICEF [32]. Along with different promotional campaigns such as the celebration of the World Breastfeeding week and intensive mass media programs contributed significantly to increase EBF practice among mothers in Bangladesh [16]. However, it seems that such promotional campigns do not contributing adequately to increase of EBF in Bangladesh in recent years as this study found a slight declined rate of EBF than the previous years.

One key finding of this study is that the prevalence of EBF practice in 2014 was lower than the prevalence of EBF in 2011, though the odds were 50% higher. This finding was consistent with the previous studies conducted in Bangladesh [27, 33]. Such a decrease could be associated with the recent societal and community-level changes in Bangladesh [32]. For instance, women's education enrollment and participation in the labor force (mostly educated women) have increased manifolds in Bangladesh [34], even much faster than the growth of male participation in the labor force. These two factors were found negatively associated with EBF practice in our analyses [27, 33]. While some studies in developing countries reported opposite results [35–37]; many other country-level studies, including Nigeria, Brazil, and Ghana [35–39], and a systematic review [40] found similar results. Along with enrolment in education and involvement in labor force, women also have to deal with several factors such as increased work pressure, travel time, and availability of breastfeeding facility in the institution and workplace. As a result, all these factors negatively affect mothers’ behaviours towards practising EBF [34, 41].

Aligned with other developing countries (e.g. Nigeria, Brazil, Ghana, and Ethiopia) private sectors including industrial and manufacturing companies are the place of 67% women's job, which are do not cater favourable environment for mothers to breastfeed their children [35–37, 40]. Factors such as long and hazardous commute to work, long working hours, inadequately equipped to offer safe spaces for mothers to breastfeed, no provision on paid breastfeeding breaks, and daycare facilities have been restricting women from continuing breastfeeding their children [42]. Although labour act recommends for a suitable room with appropriate privacy for every 40 mothers to ensure breastfeeding their child, those earlier mentioned factors are not well-addressed in the national legislation of Bangladesh [43]. However, this act is implemented for women working in the public sector [27]. Although findings of this study found a significant association between EBF and accessing intrapartum care (such as ANC), there is no provision of maternity leave in the national legislation to receive intrapartum care (such as ANC) [13, 37]. Therefore, implementation of the Labour Act regarding maternity leave as well as ensuring daycare and EBF facilities in both public and private sectors can play a crucial role to improve EBF practise in Bangladesh.

Knowledge-based awareness programs for the wider community including health care providers, community, and religious leaders, displaying posters containing awareness messages in selected areas, and trained up local health personnel up to 20 hours for making 'baby-friendly' hospitals could bring positive changes of EBF practice [43]. These are the major programs that have been launched by the GoB to ensure EBF for every child. However, such awareness programs have little impacts in increasing EBF practice among places with lower literacy rate and higher fertility rates. Areas with low literacy and higher fertility rates usually contain complex social structure with deeply-rooted misconceptions, malnourished mothers, lower use of intrapartum and postpartum care [44]. Therefore, community-specific programs such as interpersonal communication on breastfeeding and community mobilisation through mass media campaigns could be more effective in increasing the EBF practice [45]. It is also essential to ensure antenatal and delivery care and postnatal care services utilisation, particularly in communities with lower literacy and higher fertility rate. This consequently will contribute to increasing the EBF practice [28, 31, 39].

Importantly, this study also found that increased use of ANC service in community-level was more influential in increasing EBF than the individual level. Two different mechanisms (direct and indirect) might lead such differences of association. In the direct mechanism, healthcare providers might have provided appropriate information to women about the importance of EBF practice and use of subsequent maternal healthcare services (delivery care, postnatal care which further increase EBF). Moreover, in the indirect mechanism, women who have received services might share their knowledge and experiences with other women which could contribute positively in the increased use of EBF. Usually, people in Bangladesh live in clusters which are formed by education and socio-economic status. Therefore, women in a particular cluster use certain healthcare services provided by medical health workers consistently, while other groups of women might not have access to similar services. This contributes to the practice and non-practice of EBF among women from different socio-economic backgrounds. This could be an essential explanation of regional level differences of EBF practice that revealed in this analysis, and this analysis is consistent with previously published researches as well [13, 37]. However, this relationship might not be true for postnatal health care services, where this study found no evidence of an association between community-level postnatal care service use and EBF practice. The possible reason for such contradiction is that accessing postnatal care is more common in Bangladesh among women who live in urban areas, have higher education and are engaged in jobs [27]. These factors had a negative association with EBF practice in this study as well as other studies in LLMICs [20, 27, 46]. This could be because of their increasing job engagements and not supportive breastfeeding environment in the workplace [2]. Therefore, interventions to ensure services in communities, particularly in the communities with low or non-use of intrapartum and postpartum services and make breastfeeding supportive environment in the workplace, could contribute to the substantial increase in EBF practise in Bangladesh.

Use of high-quality, nationally representative data from two household surveys was the primary strength of this study. Moreover, appropriate statistical adjustments for the survey design and modelling for the confounding effects make the findings of this study more reliable. However, the primary source of limitation was recall bias. Data analysed in this study were retrospective response for the women having at least one child six months or younger. Therefore, sometimes women might not had been in a position to recall correctly all the events that took place in the time of breastfeeding initiation, intrapartum, and postpartum care use.

## Conclusion

Around 61% of the total children in Bangladesh exclusively breastfeed up to six months. Higher education, participation in formal jobs, and not accessing ANC were the individual-level factors associated with the decreasing of odds of EBF practice among mothers. Place of residence, moderate level of education, higher fertility rate, higher utilisation of ANC and delivery health care services at the community-level were found positively associated with the practice of EBF. Ensuring appropriate environment at workplace for breastfeeding mothers in favouring the continuation of their EBF practice is necessary. Furthermore, the implementation of relevant labour acts regarding maternity care in both public and private sectors may contribute to substantial increase in EBF practice in Bangladesh. Outreach programs to ensure healthcare service utilisation throughout pregnancy and delivery, and increase individuals- as well as community-level awareness of EBF practice, are also important to increase EBF practice.

## Abbreviations

EBF: Exclusive Breastfeeding
DHS: Demographic and Health Survey
WHO: World Health Organization
BDHS: Bangladesh Demographic and Health Survey
NIPORT: National Institute of Population Research and Training
aOR: adjusted Odds Ratio
